# Provider Compliance With Guidelines for Management of Cardiovascular Risk in HIV-Infected Patients

**DOI:** 10.5888/pcd10.120083

**Published:** 2013-01-24

**Authors:** Kenneth A. Lichtenstein, Carl Armon, Kate Buchacz, Joan S. Chmiel, Kern Buckner, Ellen Tedaldi, Kathleen Wood, Scott D. Holmberg, John T. Brooks, the HOPS Investigators

**Affiliations:** Author Affiliations: Kenneth A. Lichtenstein, Kern Buckner, National Jewish Health, Denver, Colorado; Carl Armon, Kathleen Wood, Cerner Corporation, Vienna, Virginia; Kate Buchacz, Scott D. Holmberg, John T. Brooks, Centers for Disease Control and Prevention, Atlanta, Georgia; Joan S. Chmiel, Northwestern University, Feinberg School of Medicine, Chicago, Illinois; Ellen Tedaldi, Temple University School of Medicine, Philadelphia, Pennsylvania.

## Abstract

**Introduction:**

Compliance with National Cholesterol Education Program Adult Treatment Panel III (NCEP) guidelines has been shown to significantly reduce incident cardiovascular events. We investigated physicians’ compliance with NCEP guidelines to reduce cardiovascular disease (CVD) risk in a population infected with HIV.

**Methods:**

We analyzed HIV Outpatient Study (HOPS) data, following eligible patients from January 1, 2002, or first HOPS visit thereafter to calculate 10-year cardiovascular risk (10yCVR), until September 30, 2009, death, or last office visit. We categorized participants into four 10yCVR strata, according to guidelines determined by NCEP, the Infectious Disease Society of America, and the Adult AIDS Clinical Trials Group. We calculated percentages of patients treated for dyslipidemia and hypertension, calculated percentages of patients who achieved recommended goals, and categorized them by 10yCVR stratum.

**Results:**

Of 2,005 patients analyzed, 33.7% had fewer than 2 CVD risk factors. For patients who had 2 or more risk factors, 10yCVR was less than 10% for 28.2%, 10% to 20% for 18.2%, and higher than 20% for 20.0% of patients. Of patients eligible for treatment, 81% to 87% were treated for elevated low-density lipoprotein cholesterol/non–high-density lipoprotein cholesterol (LDL-C/non–HDL-C), 2% to 11% were treated for low HDL-C, 56% to 91% were treated for high triglycerides, and 46% to 69% were treated for hypertension. Patients in higher 10yCVR categories were less likely to meet treatment goals than patients in lower 10yCVR categories.

**Conclusion:**

At least one-fifth of contemporary HOPS patients have a 10yCVR higher than 20%, yet a large percentage of at-risk patients who were eligible for pharmacologic treatment did not receive recommended interventions and did not reach recommended treatment goals. Opportunities exist for CVD prevention in the HIV-infected population.

## MEDSCAPE CME

Medscape, LLC is pleased to provide online continuing medical education (CME) for this journal article, allowing clinicians the opportunity to earn CME credit.

This activity has been planned and implemented in accordance with the Essential Areas and policies of the Accreditation Council for Continuing Medical Education through the joint sponsorship of Medscape, LLC and Preventing Chronic Disease. Medscape, LLC is accredited by the ACCME to provide continuing medical education for physicians.

Medscape, LLC designates this Journal-based CME activity for a maximum of 1 **AMA PRA Category 1 Credit(s)™**. Physicians should claim only the credit commensurate with the extent of their participation in the activity.

All other clinicians completing this activity will be issued a certificate of participation. To participate in this journal CME activity: (1) review the learning objectives and author disclosures; (2) study the education content; (3) take the post-test with a 70% minimum passing score and complete the evaluation at www.medscape.org/journal/pcd (4) view/print certificate.

**Release date: January 23, 2013; Expiration date: January 23, 2014**

### Learning Objectives

Upon completion of this activity, participants will be able to:

- Assess the association between HIV infection and cardiovascular risk factors
- Analyze the cardiovascular risk profile of patients with HIV in the current study
- Evaluate physician adherence to cardiovascular risk treatment among patients with HIV
- Distinguish variables associated with not receiving recommended treatment for cardiovascular risk factors

**EDITORS**

Camille Martin, editor; Ellen Taratus, editor, *Preventing Chronic Disease*. Disclosure: Camille Martin and Ellen Taratus have disclosed no relevant financial relationships.

**CME AUTHOR**

Charles Vega, MD, Associate Professor and Residency Director, Department of Family Medicine, University of California-Irvine, Irvine. Disclosure: Charles P. Vega, MD, FAAFP, has disclosed no relevant financial relationships.

**AUTHORS AND CREDENTIALS**

Disclosures: Kenneth A. Lichtenstein, MD, has disclosed the following financial relationships: Received grants for clinical research from ViiV and Abbott. He serves on advisory boards for Bristol-Myers Squibb and ViiV; Carl Armon, PhD; Kate Buchacz, PhD; Joan S. Chmiel, PhD, have disclosed no relevant financial relationships; Kern Buckner, MD, has disclosed the following financial relationships: Serves on an advisory board for Genesee BioMedical and has intellectual property with that company. He serves on the Board of Directors and has intellectual property with Wireless Medical, Inc. He also is on the Speaker Bureau for Boehringer Ingelheim Pharmaceuticals, Inc.; Ellen Tedaldi, MD, receives research support from Merck; Kathleen Wood, BSN; Scott D. Holmberg, MD; John T. Brooks, MD, have disclosed no relevant financial relationships.

Affiliations: Kenneth A. Lichtenstein, Kern Buckner, National Jewish Health, Denver, CO; Carl Armon, Kathleen Wood, Cerner Corporation, Vienna, VA; Kate Buchacz, Scott D. Holmberg, John T. Brooks, Centers for Disease Control and Prevention, Atlanta, GA; Joan S. Chmiel, Northwestern University, Feinberg School of Medicine, Chicago, IL; Ellen Tedaldi, Temple University School of Medicine, Philadelphia, PA.

## Introduction

Compliance with guidelines established in 2001 by the National Cholesterol Education Program Adult Treatment Panel III (NCEP) for primary and secondary prevention of myocardial infarction significantly reduces incident cardiovascular events (1–5). In 2003, the Infectious Disease Society of America and the Adult AIDS Clinical Trials Group published a modification of the 2001 NCEP guidelines to address management of cardiovascular risk (CVR) in HIV-1 infected people (6). In addition to describing management of traditional CVR factors, the modification included recommendations for altering antiretroviral therapy (ART). We analyzed the degree to which the modified NCEP guidelines have been followed by physicians in the HIV Outpatient Study (HOPS) cohort and the effectiveness of these interventions in reaching NCEP treatment goals designed to reduce incident cardiovascular events.

## Methods

### HIV Outpatient Study cohort

The HOPS is an ongoing, prospective, observational cohort study of HIV-infected patients receiving care in 10 participating HIV clinics (4 universities, 2 public clinics, 4 private clinics) in 8 US cities (Chicago, Illinois; Denver, Colorado; Stony Brook, New York; Oakland/San Leandro, California; Philadelphia, Pennsylvania; Tampa, Florida; and Washington, DC) since March 1993. The cohort represents a convenience sample of patients receiving HIV care at the sites; enrollment varies by clinic size. Patient data, including sociodemographic characteristics, symptoms, signs, diagnoses, treatments, and laboratory values, are abstracted from medical charts and entered into an electronic database by trained staff. Data are reviewed for quality and analyzed centrally. The HOPS protocol is reviewed and approved annually by the Centers for Disease Control and Prevention and each local site’s institutional review board. This analysis used HOPS data from January 1, 2002, through September 30, 2009.

### Study population

Entry criteria for participation in HOPS are documented HIV infection and informed consent to participate in the study. To assess achievement of treatment goals based on the 2001 NCEP guidelines, we included HOPS patients active on or after January 1, 2002, to allow clinicians the opportunity to respond to the 2001 guidelines. We defined “active” patients as those who had attended 2 or more office visits since January 1, 2002. The categorization of patients by their baseline 10-year CVR (10yCVR) required that eligible patients have had 2 or more blood pressure readings recorded and at least 1 fasting lipid panel obtained as baseline measurements any time between 12 months before and up to 9 months after the baseline date, and at least 1 year after baseline. Patients were followed until September 30, 2009, death, or last office visit.

We assessed whether the percentages of patients treated per modified NCEP guidelines and achieving recommended goals at any time during the follow-up period differed across progressively higher categories of baseline 10yCVR and according to key baseline demographic characteristics. We also calculated rates of incident cardiovascular events per 100 person-years of observation by 10yCVR group for descriptive purposes because of the small number of cardiovascular events.

### Classification of patients into 10yCVR categories

We categorized HOPS patients by baseline 10yCVR into 4 categories per NCEP guidelines (2). These guidelines categorize risk using a combination of the Framingham 10-year absolute risk for developing coronary heart disease (CHD) and conditions defined as CHD equivalents (eg, diabetes mellitus [diabetes], prior cardiovascular event). A Framingham risk calculation was not performed in patients who had low risk (ie, ≤1 major risk factor) and for whom 10yCVR was less than 10%. A Framingham risk calculation was performed among patients with 2 or more major risk factors, and they were categorized as follows: those with a 10yCVR of less than 10% were at moderate risk, those with a 10yCVR of 10% to 20% were at moderately high risk, and those with either CHD, a CHD equivalent, or a 10yCVR higher than 20% were at high risk.

Major risk factors for CHD included cigarette smoking (past or current), hypertension (systolic/diastolic blood pressure ≥140/90 mm Hg; or for patients diagnosed with diabetes or chronic renal insufficiency, ≥130/80 mm Hg; or prescription of antihypertensive therapy with a diagnosis of hypertension, regardless of blood pressure), elevated serum low-density lipoprotein cholesterol (LDL-C), serum high-density lipoprotein cholesterol (HDL-C) less than 40 mg/dL for men and less than 50 mg/dL for women, a family history of premature CHD (first-degree male relative aged <55 y, first-degree female relative aged <65 y), and older age (men, ≥45 y; women, ≥55 y). CHD equivalents are conditions that pose a CHD event risk equal to the risk for having a new CHD event in people with established CHD; these include known noncardiac vascular disease (peripheral arterial disease, abdominal aortic aneurysm, and symptomatic carotid artery disease) and diabetes. We compared the 2,005 patients with those who were excluded because of missing 1 or more required lipid or blood pressure readings.

Clinical interventions for lipid and blood pressure management are made according to 10yCVR risk category. A switch in ART at any time during the study period from a regimen associated with lipid abnormalities or insulin resistance to a regimen less likely to have these associations was considered an appropriate intervention (7–14).

### NCEP treatment goals and thresholds for medical intervention

Conditions for modification are 1) LDL-C in people with triglyceride levels ≤200 mg/dL and 2) non–HDL-C (total cholesterol minus HDL-C) when triglycerides exceed 200 mg/dL. Recommended values of LDL-C and non–HDL-C at which to initiate therapeutic lifestyle changes and/or drug therapy, as well as target values (goal), differ by each of the 4 NCEP-defined 10yCVR categories (Table 1). We included 3 additional conditions for modification to reduce CVR: low HDL-C, hypertriglyceridemia, and hypertension. We defined the HDL-C value below which to initiate lipid-modifying therapy and at or above which we considered goal to have been achieved as 40 mg/dL in men and 50 mg/dL in women. We also defined a triglyceride level of ≥500 mg/dL (the level at which triglycerides adversely affect LDL-C metabolism) as the level at which medical intervention should be initiated, with a triglyceride level of less than 500 mg/dL as goal. We considered treatment of triglycerides between 200 mg/dL and 499 mg/dL as optional. Treated hypertensive patients achieved goal if blood pressure was less than 140/90 mm Hg (or <130/80 mm Hg with concurrent diabetes or chronic renal insufficiency).

**Table 1:** Low-Density Lipoprotein Cholesterol (LDL-C) Goals, by Cardiovascular Risk (CVR) Category, According to the 2001 National Cholesterol Education Program Adult Treatment Panel III (NCEP) Guidelines When Serum Triglycerides Values Are <200 mg/dL^a^

CVR Category	LDL-C Level at Which to Initiate Therapeutic Lifestyle Changes, mg/dL	LDL-C Level at Which to Consider Lipid-Lowering Therapy, mg/dL	LDL-C Goal, mg/dL
Low risk, ≤1 major risk factor (10yCVR <10%)	≥160	≥190	<160
Moderate risk, ≥2 major risk factors (10yCVR <10%)	≥130	≥160	<130
Moderately high risk, ≥2 major risk factors (10yCVR 10%–20%)	≥130	≥130	<130
High risk, ≥2 major risk factors or CHD equivalent or (10yCVR >20%)	≥100	≥130	<100

Abbreviations: CHD, coronary heart disease (coronary heart disease or coronary heart disease equivalent per NCEP guidelines); HDL-C, high-density lipoprotein cholesterol.

a When serum triglycerides are ≥ 200 mg/dL, non-HDL-C goals are used instead of LDL-C goals. Non-HDL-C goals are 30 mg/dL above the LDL-C goals shown in the table.

We defined the presence of metabolic syndrome as having at least 3 of the following: fasting triglycerides of at least 150 mg/dL, low HDL-C, fasting blood glucose of at least 110 mg/dL, systolic blood pressure of at least 130 mm Hg or diastolic blood pressure of at least 85 mm Hg, and a body mass index (BMI) of at least 28.9 kg/m^2^ for men or 24.9 kg/m^2^ for women. 

We defined appropriate treatment as prescription of a 3-hydroxy-3-methylglutaryl-CoA (HMG CoA) reductase inhibitor (statin), with or without ezetimibe, or bile acid sequestrants for elevated LDL-C/non-HDL-C (Table 1); of nicotinic acid, documentation of exercise, or both for low HDL-C; of fibrates or omega-3 fatty acids for hypertriglyceridemia, and of antihypertensives for hypertension. However, data on initiation of therapeutic lifestyle changes such as increased exercise were not systematically collected in this cohort. We also considered changes in ART regimens as appropriate treatment.

### Statistical analyses

Summaries of descriptive data, univariate analyses, and Cochrane–Armitage tests for trend were conducted with SAS version 9.2 (SAS Institute, Inc, Cary, North Carolina). We compared the distributions of categorical variables using a likelihood ratio, continuity-adjusted χ^2^ test, or Fisher exact test, and we compared the distributions of continuous variables with a Wilcoxon rank-sum test for 2-group comparisons. We used a χ^2^ test and Kruskal–Wallis test for comparisons of more than 2 groups. We computed incidence rates of cardiovascular events per 100 person-years of observation for each 10yCVR category and compared them across 10yCVR categories using StatCalc (EpiInfo 2000, Centers for Disease Control and Prevention, Atlanta, Georgia). Proportion confidence intervals were calculated using mid-*P* exact confidence limits (15). Statistical associations with 2-sided *P* values less than .05 were considered significant.

## Results

Our study population of 2,005 HOPS patients (baseline median age, 42 y; median CD4+ T cell count, 395 cells/mm^3^) was predominantly male (76%) and racially/ethnically diverse (52% non-Hispanic white, 33% non-Hispanic black, and 12% Hispanic); 55% of patients were current or former tobacco users at baseline. Seventeen percent of patients had no ART exposure, 77.7% had exposure to highly active ART (HAART) (45.3% had prior mono- or dual-antiretroviral [ARV] exposures; 32.4% had HAART only), and 5.1% were classified as having unknown or missing data on ARV exposure. Of the 2,005 HOPS patients analyzed, 675 were categorized at baseline as low risk, 565 as moderate risk, 365 as moderately high risk, and 400 as high risk. Median follow-up after baseline was approximately 5.5 years and did not differ significantly across 10yCVR categories (Table 2).

**Table 2:** Characteristics of Patients (N = 2,005), by 10-Year Cardiovascular Risk (CVR) Category, HIV Outpatient Study (HOPS), January 2002–September 2009

Characteristic^a^	10-Year CVR Category					*P* Value^b^

	Overall (N = 2,005)	Low Risk (<10% Risk), ≤1Risk Factor (n = 675)	Moderate Risk (<10% Risk), ≥2 Risk Factors (n = 565)	Moderately High Risk (10%–20% Risk), ≥2 Risk Factors (n = 365)	High Risk (>20% Risk), ≥2 Risk Factors (n = 400)	

Baseline						
**Age, y, median**	42	38	41	47	48	<.001
**Male sex**	1,532 (76)	451 (67)	405 (72)	350 (96)	326 (82)	<.001
**Race/ethnicity**						
White, non-Hispanic	1,048 (52)	304 (45)	276 (49)	252 (69)	216 (54)	<.001
Black, non-Hispanic	657 (33)	251 (37)	199 (35)	73 (20)	134 (34)	
Hispanic	239 (12)	93 (14)	71 (13)	31 (8)	44 (11)	
Other	61 (3)	27 (4)	19 (3)	9 (2)	6 (2)	
**Year of HOPS entry**						
1998 or earlier	856 (43)	247 (37)	210 (37)	176 (48)	223 (56)	<.001
1999–2005	1,149 (57)	428 (63)	355 (63)	189 (52)	177 (44)	
**Private insurance**	1,147 (57)	420 (62)	293 (52)	233 (64)	201 (50)	<.001
**History of IDU**	227 (11)	37 (5)	90 (16)	38 (10)	62 (16)	<.001
**Prior AIDS-defining illness**	728 (36)	209 (31)	204 (36)	139 (38)	176 (44)	<.001
**Cell count/viral load**						
Nadir CD4+ T cell count <200 cells/mm^3^	1,016 (51)	314 (47)	296 (52)	191 (52)	215 (54)	.06
Nadir CD4+ T cell count, cells/mm^3^, median	197	218	187	190	180	.27
CD4+ T cell count, cells/mm^3^, median	395	396	358	401	415	.002
Peak viral load, copies/mL, median	5,985	7,790	19,712	1,911	2,384	<.001
Viral load, copies/mL, median	419	745	908	200	200	<.001
Viral load <400 copies/mL	988 (49)	305 (45)	244 (43)	211 (58)	228 (57)	<.001
**Alcohol use, drinks/week**						
Missing information	127 (6)	49 (7)	32 (6)	18 (5)	28 (7)	<.001
0	977 (49)	322 (48)	284 (50)	146 (40)	225 (56)	
<7	689 (34)	242 (36)	192 (34)	144 (39)	111 (28)	
7–14	124 (6)	40 (6)	30 (5)	31 (8)	23 (6)	
>14	88 (4)	22 (3)	27 (5)	26 (7)	13 (3)	
**BMI, kg/m^2^**						
Missing information	120 (6)	47 (7)	36 (6)	21 (6)	16 (4)	.005
≤25	909 (45)	331 (49)	261 (46)	160 (44)	157 (39)	
>25	976 (49)	297 (44)	268 (47)	184 (50)	227 (57)	
**Hypertension**	1,036 (52)	112 (17)	356 (63)	230 (63)	338 (85)	<.001
**Diabetes mellitus**	190 (9)	0	0	0	190 (48)	<.001
**History of tobacco use**	1,108 (55)	164 (24)	348 (62)	296 (81)	300 (75)	<.001
**Blood lipid panel measurement**						
Total cholesterol, mg/dL, median	204	197	181	226	238	<.001
LDL cholesterol, mg/dL, median	105	108	94	114	107	<.001
HDL cholesterol, mg/dL, median	37	44	35	35	35	<.001
Non-HDL cholesterol, mg/dL, median^c^	164	151	142	186	199	<.001
Triglycerides, mg/dL, median	163	126	162	200	225	<.001
Metabolic syndrome^d^	516 (26)	85 (13)	157 (28)	103 (28)	171 (43)	<.001
**Antiretroviral therapy**						
Efavirenz	452 (23)	157 (23)	136 (24)	66 (18)	93 (23)	.14
Nevirapine	213 (11)	65 (10)	63 (11)	38 (10)	47 (12)	.70
Protease inhibitor, unboosted	222 (11)	62 (9)	72 (13)	41 (11)	47 (12)	.23
Protease inhibitor, boosted	626 (31)	205 (30)	171 (30)	126 (35)	124 (31)	.51
Zidovudine or Stavudine	775 (39)	233 (35)	230 (41)	143 (39)	169 (42)	.043
Didanosine	273 (14)	78 (12)	72 (13)	62 (17)	61 (15)	.07
Abacavir	443 (22)	139 (21)	132 (23)	81 (22)	91 (23)	.68
Tenofovir	561 (28)	174 (26)	181 (32)	92 (25)	114 (29)	.055
**Lipid-lowering agents**						
Statin drug/ezitimibe	245 (12)	42 (6)	31 (5)	49 (13)	123 (31)	<.001
Fibrate	116 (6)	13 (2)	16 (3)	26 (7)	61 (15)	<.001
Fish oil	17 (1)	4 (1)	3 (1)	2 (1)	8 (2)	.09

**Follow-Up**						

**Follow-up after baseline, y, median**	5.5	5.5	5.5	5.8	5.1	.32
**Lipid-lowering agents**						
Statin drug/ezitimibe	562 (28)	119 (18)	109 (19)	125 (34)	209 (52)	<.001
Fibrate	235 (12)	39 (6)	53 (9)	54 (15)	89 (22)	<.001
Fish oil	139 (7)	25 (4)	42 (7)	33 (9)	39 (10)	<.001
**ARV regimen changes^e^**	1,353 (67)	442 (65)	380 (67)	260 (71)	271 (68)	.30
**Antihypertensives**	633 (32)	52 (8)	212 (38)	136 (37)	233 (58)	<.001
**Incident CVD**	148 (7)	14 (2)	27 (5)	45 (12)	62 (16)	<.001

Abbreviations: IDU, intravenous drug use; AIDS, acquired immunodeficiency syndrome; BMI, body mass index; LDL, low-density lipoprotein; HDL, high-density lipoprotein; ARV, antiretroviral.

a Values presented as n (%) unless otherwise indicated.

b χ^2^ test for binary variables or Kruskal–Wallis test for continuous variables.

c Non-HDL cholesterol equals total cholesterol minus HDL cholesterol.

d Defined as the presence of any 3 of the following 5 risk factors: hypertension (≥130 mm Hg over ≥85 mm Hg), elevated triglycerides (≥150 mg/dL), low HDL cholesterol (<40 mg/dL for men, <50 mg/dL for women), fasting glucose ≥110 mg/dL, abdominal obesity (BMI ≥28.9 kg/m^2^ for men and ≥24.9 kg/m^2^ for women).

e Treatment of either high LDL-cholesterol/non-HDL-cholesterol or hypertriglyceridemia can include the following ARV changes: Zidovudine/Stavudine to Abacavir or Tenofovir; Abacavir to Tenofovir; boosted protease inhibitor to unboosted protease inhibitor; any protease inhibitor to Efavirenz or Nevirapine; Efavirenz to Nevirapine; boosted Indinavir or Lopinavir to boosted Darunavir, Atazanavir, fos-Amprenavir, or Saquinavir/Fortovase.

At baseline, as 10yCVR increased, the percentage of people with the following characteristics increased significantly: those with a diagnosis of either hypertension or metabolic syndrome; median serum total cholesterol and triglycerides; and those prescribed lipid-lowering agents. Median serum HDL-C decreased significantly with increasing 10yCVR categories, whereas LDL-C, the distribution by race/ethnicity, and baseline use of ART did not vary consistently (Table 2).

Excluded patients were significantly younger (median age, 40 vs 42 y); more likely to be male (82.0% vs 76.4%); more likely to be white (56.2% vs 52.3%); more likely to be privately insured (65.5% vs 57.2%); and less likely to have had an AIDS-defining illness (22.0% vs 36.3%), diabetes (4.3% vs 9.5%), hypertension (27.5% vs 51.7%), or BMI higher than 30 kg/m^2^ (12.7% vs 19.4%); and more likely to have unknown or missing ARV history (17.0% vs 5.1%) at baseline.

As 10yCVR increased, the percentage of people with each cardiovascular condition of interest increased (Table 3). The percentage of patients treated among those patients with each condition of interest also increased; the highest percentage was treated for high LDL-C and hypertriglyceridemia. However, the percentage of patients treated according to modified NCEP guidelines and who met guideline-defined goals at any time during the follow-up period for each condition decreased across each of the 4 CVR categories, particularly in the high-risk group.

**Table 3:** Treatment of Patients (N = 2,005) for High LDL-C/Non-HDL-C, Low HDL-C, Hypertriglyceridemia, and Hypertension, by 10-Year Cardiovascular Risk (CVR) Category, HIV Outpatient Study, January 2002–September 2009

Condition/CVR Category^a^	% (n/N) With Condition	% (n/N) Treated Per Guidelines^b^	% (n/N) Treated Who Met NCEP Goals^c^
**High LDL-C/non-HDL-C**	*P* < .001^d^	*P* = .68	*P* < .001
Low risk	15.9 (107/675)	81.3 (87/107)	81.6 (71/87)
Moderate risk	31.5 (178/565)	86.5 (154/178)	62.3 (96/154)
Moderately high risk	62.2 (227/365)	83.3 (189/227)	59.3 (112/189)
High risk	80.0 (320/400)	84.1 (269/320)	33.5 (90/269)
**Low HDL-C**	*P* < .001	*P* < .001	*P* = .42
Low risk	41.6 (281/675)	2.9 (8/281)	50.0 (4/8)
Moderate risk	78.4 (443/565)	1.6 (7/443)	14.3 (1/7)
Moderately high risk	67.7 (247/365)	8.1 (20/247)	20.0 (4/20)
High risk	73.0 (292/400)	11.3 (33/292)	27.3 (9/33)
**Hypertriglyceridemia**	*P* < .001	*P* = .03	*P* = .84
Low risk	2.4 (16/675)	56.3 (9/16)	88.9 (8/9)
Moderate risk	5.8 (33/565)	90.9 (30/33)	80.0 (24/30)
Moderately high risk	12.3 (45/365)	82.2 (37/45)	78.4 (29/37)
High risk	18.3 (73/400)	87.7 (64/73)	76.6 (49/64)
**Hypertension**	*P* < .001	*P* < .001	*P* < .001
Low risk	16.6 (112/675)	46.4 (52/112)	67.3 (35/52)
Moderate risk	63.0 (356/565)	59.6 (212/356)	66.0 (140/212)
Moderately high risk	63.0 (230/365)	59.1 (136/230)	64.0 (87/136)
High risk	84.5 (338/400)	68.9 (233/338)	46.4 (108/233)

Abbreviations: LDL-C, low-density lipoprotein cholesterol; HDL-C, high-density lipoprotein cholesterol; NCEP, National Cholesterol Education Program Adult Treatment Panel III.

a See text for definitions of 10-year cardiovascular disease risk categories and definitions of high LDL-C/non-HDL-C, low HDL-C, hypertriglyceridemia, and hypertension.

b Eligible patients treated per modified NCEP guidelines within 2 years of baseline date. Treatment of either high LDL-C/non-HDL-C or hypertriglyceridemia can include the following ARV changes: Zidovudine/Stavudine to Abacavir or Tenofovir; Abacavir to Tenofovir; boosted protease inhibitor to unboosted protease inhibitor; any protease inhibitor to Efavirenz or Nevirapine; Efavirenz to Nevirapine; boosted Indinavir or Lopinavir to boosted Darunavir, Atazanavir, fos-Amprenavir, or Saquinavir/Fortovase.

c Treated patients who met modified NCEP goals after starting treatment and before the end of observation in the study.

d Likelihood ratio χ^2^ test.

**Figure 1:**
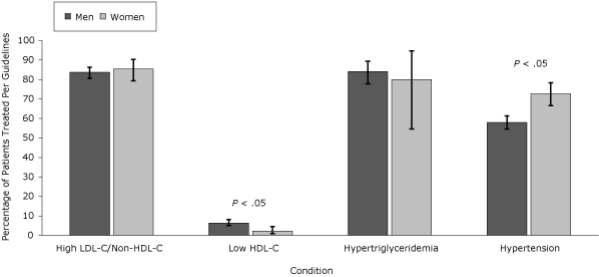
Percentage of patients with condition treated per National Cholesterol Education Program Adult Treatment Panel III (NCEP) guidelines, by sex, the HIV Outpatient Study, January 2002–September 2009. Error bars indicate 95% confidence intervals. Abbreviations: LDL-C, low-density lipoprotein cholesterol; HDL-C, high-density lipoprotein cholesterol. **Table:** 

**Figure 2:**
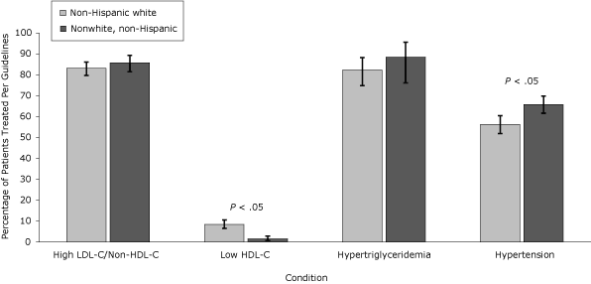
Percentage of patients with condition treated per National Cholesterol Education Program Adult Treatment Panel III (NCEP) Guidelines, by race/ethnicity, the HIV Outpatient Study, January 2002–September 2009. Error bars represent 95% confidence intervals. Abbreviations: LDL-C, low-density lipoprotein cholesterol; HDL-C, high-density lipoprotein cholesterol. **Table:** 

**Figure 3:**
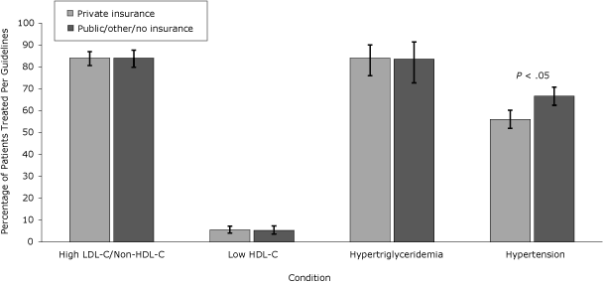
Percentage of patients with condition treated per National Cholesterol Education Program Adult Treatment Panel III (NCEP) Guidelines, by payer, the HIV Outpatient Study, January 2002–September 2009. Error bars represent 95% confidence intervals. Abbreviations: LDL-C, low-density lipoprotein cholesterol; HDL-C, high-density lipoprotein cholesterol. **Table:** 

Women, nonwhites, and patients with public insurance or no insurance were treated for hypertension per guidelines more often than men, whites, or patients with private insurance, despite a lower representation of the former patients in the higher CVR categories. Pharmacologic treatment of low HDL-C was more frequent in whites and men but overall treatment rates were low. Management of LDL-C/non-HDL-C, HDL-C, and triglycerides was similar when these groups were compared (Figures 1, 2, and 3).

Condition	Patients Successfully Treated Per NCEP Guidelines, % (95% Confidence Interval)
**High LDL-C/non-HDL-C **	
Men (n = 674)	83.7 (80.7-86.3)
Women (n = 158)	85.4 (79.3-90.3)
**Low HDL-C**	
Men (n = 956)	6.4 (5.0-8.1)
Women (n = 307)	2.3 (1.0-4.5)
**Hypertriglyceridemia**	
Men (n = 152)	84.2 (77.8-89.4)
Women (n = 15)	80.0 (54.7-94.7)
**Hypertension**	
Men (n =816)	58.0 (54.6-61.3)
Women (n = 220)	72.7 (66.6-78.3)

Condition	Patients Successfully Treated Per NCEP Guidelines, % (95% Confidence Interval)
**High LDL-C/non-HDL-C **	
Non-Hispanic white (n = 525)	83.1 (79.7-86.1)
Nonwhite, non-Hispanic (n = 307)	85.7 (81.4-89.3)
**Low HDL-C**	
Non-Hispanic white (n = 716)	8.4 (6.5-10.6)
Nonwhite, non-Hispanic (n = 547)	1.5 (0.7-2.8)
**Hypertriglyceridemia**	
Non-Hispanic white (n = 124)	82.3 (74.8-88.2)
Nonwhite, non-Hispanic (n = 43)	88.4 (78.1-95.6)
**Hypertension**	
Non-Hispanic white (n = 512)	56.3 (51.9-60.5)
Nonwhite, non-Hispanic (n = 524)	65.8 (61.7-69.8)

Condition	Patients Successfully Treated Per NCEP Guidelines, % (95% Confidence Interval)
**High LDL-C/non-HDL-C **	
Private insurance (n = 500)	84.0 (80.6-87.0)
Public/other/no insurance (n = 332)	84.0 (79.8-87.7)
**Low HDL-C**	
Private insurance (n = 732)	5.5 (4.0-7.3)
Public/other/no insurance (n = 531)	5.3 (3.6-7.4)
**Hypertriglyceridemia**	
Private insurance (n = 106)	84.0 (76.0-90.0)
Public/other/no insurance (n = 61)	83.6 (72.7-91.4)
**Hypertension**	
Private insurance (n = 546)	56.0 (51.9-60.2)
Public/other/no insurance (n = 490)	66.7 (62.5-70.8)

Incidence of cardiovascular events (per 100 person-years) increased with increasing 10yCVR category: 0.38 (14 events) in the low-risk group compared with 0.89 (27 events) in the moderate-risk group, 2.24 (45 events) in the moderately high-risk group, and 2.98 (62 events) in the high-risk group (all *P* < .01 compared with the low-risk group).

## Discussion

HIV-infected patients are at higher risk and have a higher incidence of cardiovascular events than the general public (16–22). In our cohort, 38.2% of patients were in either the moderately high-risk or high-risk categories, of whom 77.9% were current or former smokers, 74.2% had hypertension, 71.5% had baseline elevated LDL-C levels, 70.5% had low HDL-C levels, and 35.8% had metabolic syndrome. The percentage of patients with high LDL-C/non-HDL-C and triglyceride levels, low HDL-C levels, and hypertension generally increased with increasing 10yCVR category. Although most affected patients were prescribed recommended therapy for high LDL-C/non-HDL-C and hypertriglyceridemia, only half were treated for hypertension and very few were treated pharmacologically for low HDL-C. We also observed lower frequency of hypertension treatment among men, people who were white, and people who were privately insured; these lower frequencies were not explained by colinearity or differences in 10yCVR distribution.

Several studies have demonstrated increases in total cholesterol, LDL-C, HDL-C and triglycerides after initiating ART in antiretroviral-naïve patients (23–32). In untreated advanced HIV infection, levels of total cholesterol, LDL-C, and HDL-C are typically low. It is unclear to what extent the lipid elevations observed after initiating treatment of HIV infection were an adverse effect of ART versus a return to health (33). In light of the increased CVR faced by HIV-infected patients, focusing efforts on improving lipid profiles and controlling hypertension as recommended in the modified NCEP recommendations seems prudent.

HDL-C deserves additional attention. Normalizing HDL-C levels is a tertiary goal of the NCEP guidelines (34). HIV-infected patients characteristically have low HDL-C levels, and although levels rise with ART, they rarely normalize (33). We also found a high percentage of people (62% men, 65% women) in our cohort with low HDL-C measurements compared with the 1999–2002 National Health and Nutrition Examination Survey (NHANES III) population (33% men, 20% women) (35). Only 5.4% of patients in our study received pharmacologic treatment of HDL-C, of whom only 26.5% achieved target levels. Possible explanations for low compliance may include concern about side effects, pill burden, designation of HDL-C as a tertiary target, and pharmaceutical marketing emphasis on triglyceride elevations.

Although much attention has focused on the effect of ART on elevating triglyceride levels, only 8.3% of patients in our cohort had levels exceeding 500 mg/dL at baseline. Of those patients, 83.8% received triglyceride-lowering therapy and 78.6% of those patients had reductions of triglycerides to less than 500 mg/dL. Elevated triglycerides directly contribute little to increased CVR. Triglycerides exceeding 500 mg/dL interfere with the release of LDL-C from small dense atherogenic particles, resulting in deposition of these particles into atheromatous plaques (36). Statins contribute to expression of LDL-C receptors with consequent reduction of the circulating LDL-C substrate. Reducing triglycerides to less than 500 mg/dL enhances the effectiveness of statin therapy via this mechanism.

Although other cohort studies of HIV-infected patients have evaluated CVD risk factors, including 10yCVR, few to our knowledge have assessed physician adherence to guidelines for management of these conditions (18,37–39). We found that 38.2% of our study population had a 10yCVR at or higher than 10% (moderately high risk and high risk) compared with 22% to 26% in the Data Collection on Adverse Events of Anti-HIV Drugs (D:A:D) study (40). In an analysis of the Multicenter AIDS Cohort Study (MACS) and the Women’s Interagency HIV Study (WIHS), Kaplan et al defined moderate CVD risk as a 10yCVR of 15% to 25% and high CVD risk as a 10yrCVR of 25% or higher or having a diagnosis of diabetes. They found that 2% of men and 1% of women had a moderate predicted CVD risk and 17% of men and 12% of women had a high predicted CVD risk (18).

Comparing NHANES III data with those from the HOPS cohort, 30.0% versus 41.5% of people had elevated baseline LDL-C. When considering only moderately high-risk and high-risk groups, 68.0% versus 71.5% of patients had high LDL-C, of whom 25.0% versus 83.7% received treatment. Of those patients treated, 39.1% versus 59.3% in the moderately high-risk group and 22.3% versus 33.5% in the high-risk group met NCEP goals. Although comparisons of NHANES III and HOPS data are complicated by differences in study design, population sociodemographic characteristics, laboratory measurements of lipids, and ascertainment of treatment interventions, findings suggest that there is still a significant opportunity for improvement in CVD risk management in HOPS patients.

This study, which used routinely collected medical abstraction data from HIV outpatients, had several limitations. Our inclusion criteria, which required certain baseline examinations, may have enriched our analysis cohort in people whom clinicians considered to be at higher risk of CVD and who had warranted those examinations. Whether the excluded patients were considered lower risk by their physicians or had unrecognized risk factors necessarily limits the overall findings in our study. We also cannot infer reasons for lack of intervention when apparently indicated. Failure to initiate recommended interventions may have resulted from patients deferring or declining therapy, contraindications to these therapies not recorded in HOPS chart abstractions, or clinician oversight. CVD risk factor data were not systematically charted, precluding our ability to assess whether interventions resulted in sustained responses over time. We did not have adequate information on family history of cardiovascular events in first-degree relatives, a major CHD risk factor. The absence of these data would likely lead us to underestimate the true prevalence of CVR in our population. Furthermore, we did not have information on waist size for most patients. We substituted BMI at or above 28.9 kg/m^2^ for men and at or above 24.9 kg/m^2^ for women for waist size greater than 40 inches in men and greater than 35 inches in women, when measured, for our estimation of metabolic syndrome, guided by findings from prior studies in HIV cohorts (33,41). The data on pharmacotherapeutic treatments may have been recorded incompletely, and patients may have received treatments outside of the HOPS clinics (eg, cardiology specialist consultations) that were also not captured by chart abstraction. Finally, we could not account for therapeutic lifestyle change interventions such as intentional smoking cessation or weight loss, improved diet, or increased exercise; these data are not collected systematically in the HOPS.

Our findings suggest that HOPS participants who comprise a heterogeneous convenience sample of HIV-infected patients seen at 10 HIV specialty clinics in the United States have significantly greater CVD risk than both the general US population and participants in other HIV cohorts. A large percentage of at-risk patients who were eligible for pharmacologic treatment did not receive recommended interventions and did not reach recommended treatment goals. As HIV-infected patients live longer, it is imperative that clinicians minimize CVD risk by optimizing patients’ lipid profiles and blood pressure and helping patients who use tobacco products to quit. Intensified educational efforts are needed and further research is warranted to understand the barriers to initiating recommended interventions and to achieving recommended treatment goals that are unique to HIV-infected patients.

## Figures and Tables

**Table:** 

**1. The activity supported the learning objectives.**				
**Strongly Disagree**				**Strongly Agree**
1	2	3	4	5
**2. The material was organized clearly for learning to occur.**				
**Strongly Disagree**				**Strongly Agree**
1	2	3	4	5
**3. The content learned from this activity will impact my practice.**				
**Strongly Disagree**				**Strongly Agree**
1	2	3	4	5
**4. The activity was presented objectively and free of commercial bias.**				
**Strongly Disagree**				**Strongly Agree**
1	2	3	4	5

## Post-Test Information

To obtain credit, you should first read the journal article. After reading the article, you should be able to answer the following, related, multiple-choice questions. To complete the questions (with a minimum 70% passing score) and earn continuing medical education (CME) credit, please go to http://www.medscape.org/journal/pcd. Credit cannot be obtained for tests completed on paper, although you may use the worksheet below to keep a record of your answers. You must be a registered user on Medscape.org. If you are not registered on Medscape.org, please click on the "Register" link on the right hand side of the website to register. Only one answer is correct for each question. Once you successfully answer all post-test questions you will be able to view and/or print your certificate. For questions regarding the content of this activity, contact the accredited provider, CME@medscape.net. For technical assistance, contact CME@webmd.net. American Medical Association's Physician's Recognition Award (AMA PRA) credits are accepted in the US as evidence of participation in CME activities. For further information on this award, please refer to http://www.ama-assn.org/ama/pub/category/2922.html. The AMA has determined that physicians not licensed in the US who participate in this CME activity are eligible for **AMA PRA Category 1 Credits™**. Through agreements that the AMA has made with agencies in some countries, AMA PRA credit may be acceptable as evidence of participation in CME activities. If you are not licensed in the US, please complete the questions online, print the AMA PRA CME credit certificate and present it to your national medical association for review.

## Post-Test Questions

### Article Title: Provider Compliance with Guidelines for Management of Cardiovascular Risk in Patients Infected With the Human Immunodeficiency Virus

**CME Questions**

1. You are seeing a 50-year-old man with a 10-year history of HIV infection. His body mass index is 29 kg/m^2^, and you are concerned with his cardiovascular risk. Which of the following statements regarding cardiovascular risk factors among patients with HIV is most accurate?
  - Persons with HIV infection are generally at the same risk of cardiovascular disease as persons without HIV
  - LDL-C is typically high among patients with HIV who have not received antiretroviral therapy (ART)
  - ART increases levels of multiple classes of serum lipids
  - Triglycerides are the most important class of lipids in promoting cardiovascular disease among patients with HIV
2. What should you consider regarding the distribution of cardiovascular risk among patients in the current study?
  - More than 90% of participants had 2 or more cardiovascular risk factors
  - One fifth of participants had a 10-year cardiovascular risk (10yCVR) of over 20%
  - Hypertriglyceridemia was the most common cardiovascular risk factor
  - High LDL-C was the most common cardiovascular risk factor
3. Upon chart review, the patient you are seeing has multiple cardiovascular risk factors. Which of the following risk factors was ***LEAST*** likely to be treated in the current study
  - High LDL-C
  - Low HDL-C
  - Hypertriglyceridemia
  - Hypertension
4. The patient has hypertension. Which of the following variables was a risk factor for ***not*** receiving appropriate hypertension treatment in the current study
  - Female sex
  - Nonwhite race
  - Borderline blood pressure value
  - Private health insurance

**Evaluation**
